# Serum inflammatory and oxidative stress biomarkers levels are associated with pain intensity, pressure pain threshold and quality of life in myofascial pain syndrome

**DOI:** 10.1186/s13104-020-05352-3

**Published:** 2020-11-07

**Authors:** Seyed Kazem Shakouri, Neda Dolatkhah, Sepideh Omidbakhsh, Alireza Pishgahi, Maryam Hashemian

**Affiliations:** 1 Physical Medicine and Rehabilitation Research Center, Aging Research Institute, Tabriz University of Medical Sciences, Tabriz, Iran; 2 Department of Physical Medicine and Rehabilitation, Faculty of Medicine, Tabriz University of Medical Sciences, Tabriz, Iran; 3 Department of Biology, School of Art and Science, Utica College, Utica, NY USA; 4 Physical Medicine and Rehabilitation Research Center, Emam Reza Hospital, Golgasht, Azadi Ave, Tabriz, Iran

**Keywords:** Myofascial pain syndrome (MPS), Inflammation, Oxidative stress, Quality of life

## Abstract

**Objectives:**

We aimed to determine the serum concentrations of some inflammatory and oxidative stress biomarkers in relation with pain intensity and quality of life in patients with myofascial pain syndrome (MPS) compared to healthy controls. This study is a case–control study. The participants were selected from MPS patients who referred to rehabilitation outpatient clinics of the Tabriz University of Medical Sciences, Iran.

**Results:**

Serum hs-CRP (4.68 ± 4.36 vs. 2.92 ± 4.55 g/mlµ respectively, p = 0.011), phospholipase A2 (PLA2) (6.81 ± 2.22 vs. 4.73 ± 2.97 pg/ml respectively, p < 0.001) and malondialdehyde (MDA) (2.63 ± 0.71 vs. 1.98 ± 0.90 nmol/ml respectively, p < 0.001) levels were significantly higher and serum total antioxidant capacity (TAC) (2.46 ± 0.49 vs. 2.83 ± 0.82 mmol/L respectively, p = 0.011) and superoxide dismutase (SOD) (78.89 ± 37.93 vs. 154.25 ± 115.93 U/ml respectively, p < 0.001) levels were significantly lower in the MPS patients compared to healthy controls. Serum high-sensitivity C-reactive protein (hs-CRP) level was significantly and positively associated with resting (r = 0.349, p = 0.019), activity (r = 0.295, p = 0.049) and night pain (r = 0.304, p = 0.043) intensities, pressure pain threshold (PPT) (r = 0.210, p = 0.047) and pain duration (r = 0.283, p = 0.007). Serum TAC level was significantly and negatively associated with resting pain intensity (r = −0.312, p = 0.037). Some scales and subscales of quality of life were positively correlated with serum TAC level and negatively associated with serum hs-CRP and PLA2 levels.

## Introduction

Myofascial pain syndrome (MPS) is a chronic painful dysfunction that can distress any striated muscle in the human body [1]. MPS is a very common and costly disorder affecting society, and its prevalence has been reported currently from 30 to 85% [2, 3]. The exact pathophysiologic mechanism of MPS is still poorly understood, yet new researches are enlightening the mechanisms that could have a noteworthy effect on the way this disorder is established and managed [4]. Biochemical changes have been studied to some extent in MPS. However, more in-depth studies, especially on sub-acute inflammation and oxidative stress in relation with clinical presentation, are required to establish their potential role in the diagnosis and treatment of disease [5–7]. Additionally, pain is frequently accompanying with other complaints such as depression, mental stress, anxiety, and, subsequently decreased quality of life [8] and there is very limited evidence regarding the association between inflammatory and oxidative stress indices and quality of life in the scientific literature especially in MPS patients.

The primary aim of this study was to compare serum concentrations of important inflammatory biomarkers and oxidative stress between primary MPS patients and age and body mass index (BMI) matched healthy controls. The secondary aim of this study was to determine the correlation between these biomarkers with clinical findings, and quality of life exists.

## Main text

### Methods

#### Study population

The present study was a case–control study. Patients with the primary MPS attending the university rehabilitation outpatient clinics and satisfying inclusion criteria [(age between 18 and 60 years, patients with persisting neck or shoulder pain for ≥ 3 months, MTrPs in one or more of the trapezius, the infraspinatus, and/or the levator scapulae muscles, visual analog scale (VAS) > 4) and normal neurologic examination], were involved via simple random sampling from October 2018 to May 2019. The exclusion criteria were fibromyalgia, cervical radiculopathy, metabolic diseases like hypothyroidism and diabetes mellitus, and previous injection for MPS treatment within the last 6 months before the enrollment.

The control group was a sample of healthy subjects, matched for age (± 2 years) and BMI selected randomly from the other outpatient clinics of the same university. The exclusion criteria were the presence of systemic diseases, chronic pain, or psychiatric disorders. The ratio of the case to the control group was 1:1. The study was carried out following the principles of the Declaration of Helsinki. Written informed consent was achieved from all participants.

#### Sample size

Based on a previous study by Koca et al. [9] and TAC level of 0.93 ± 0.11 (mmol Trolox equivalent/l) in subjects in the case group and 1.05 ± 0.20 (mmol Trolox equivalent/l) in subjects in the control group, power as 80%, type of test as two-sided and the probability of incomplete data as 15%, a sample size of 45 in each group and 90 in total was estimated.

### Measurements

#### Demographics and medical information

The demographic data of participants were gathered through face to face interviews by a general characteristics questionnaire containing demographic and medical information. BMI was calculated by dividing the weight (kg) by the square of height (m^2^) [10]. The physical activity of the study participants was evaluated through the short-form International Physical Activity Questionnaire (IPAQ) [11]. Three categories of physical activity were proposed: low, moderate, and high [12].

#### Visual analogue scale (VAS)

VAS was used to measure the pain observed by the subjects in the last 24 h through a 0–10 cm scale. Each patient was instructed to represent their perceived resting pain, night pain, and pain intensity with the activity which ranged from ‘0’ (no pain) to ‘10’ cm (worst imaginable pain). The VAS is a simple and commonly applied method for the evaluation of pain intensity [13]. This rating scale has been confirmed to have satisfactory psychometric powers to be used in chronic pain studies, and also to be more appropriate when the greatest consistency is needed [14].

#### Pressure pain threshold (PPT)

The least force exerted which persuaded pain was inspected using PPT through the pressure algometry method. Estimation precision was distinguished at 1 kg/cm^2^. An electronic pressure algometry device was placed perpendicularly on the location of the trigger point to increasing compression pressure against the muscle under examination by 1 kg/cm^2^/s [15].

#### Quality of life

The validated Persian version of the 36-item questionnaire SF-36 was used to evaluate the quality of life in participants [16].

#### Biochemical analysis

Serum high-sensitive C-reactive protein (hs-CRP) (μg/mL) was measured by the Monobind hs-CRP Elisa kit. The Luminex-based immunoassay using the Serum Phospholipase A2 kit (Nevandsalamat) was used to measure phospholipase A2 (PLA2) as described by Hsu et al. [17]. Measurement of serum TAC concentration was performed using the Naxifer™-Total Antioxidant Capacity Assay Kit-TAC Colorimetric Assay Kit [18]. Serum Malondialdehyde (MDA) was measured using Nalondi™-Lipid Peroxidation Assay Kit-MDA. Serum superoxide dismutase (SOD) levels were measured by colorimetric method using an ELISA kit (RANDOX, Antrim, North Ireland UK) according to the manufacturers’ instruction.

#### Statistical analysis

All statistical analyses were performed using the statistical package SPSS (version 19.0, SPSS Inc., Chicago, IL). An independent sample t-test or Mann–Whitney U test was done to inspect the differences in inflammatory markers and oxidative stress indices between patients with MPS and non-MPS controls. Unadjusted linear regression was run to assess the association between inflammation and oxidative stress biomarkers with pain intensity and quality of life. A P-value ≤ 0.05 was considered statistically significant.

### Results

A total of 96 patients with MPS were evaluated for eligibility. Of these, 11 were excluded due to the disagreement of participation in this study or lack of inclusion criteria. Finally, 85 participants were studied in this. Distribution of demographic variables like age, weight and BMI, serum levels of biomarkers, and subscales of quality of life are presented in Tables 1 and 2. There were no significant differences in demographic findings and also anthropometric indices between case and control group (P > 0.05). Twenty, 13, and 10 patients in the case group had MTrPs in the trapezius muscle, trapezius + infraspinatus muscles, and trapezius + levator scapulae muscles, respectively. The mean duration of symptoms in patients was 9.93 ± 4.36 months. The intensity of resting pain, nocturnal pain, and pain during the activity were 5.51 ± 2.11, 5.11 ± 3.29, and 7.96 ± 1.74, respectively. The PPT was 2.19 ± 0.65. The concentration of hs-CRP and PLA2 and MDA were significantly higher in MPS patients compared with those of healthy controls. The levels of SOD were significantly lower in patients with MPS compared with those of healthy controls. All scales and subscale of quality of life were significantly lower in MPS patients compared with those of healthy controls, except Physical function (PF), Energy/fatigue (EF), and Emotional wellbeing (EW).

**Table 1:** Distribution of demographic characteristics among the study participants

Variable	Case group (n = 43)	Control group (n = 42)	P value^c^
Age (year)	9.35^a^ 36.44 ±	37.13 ± 8.47	0.715
Weight (kg)	71.58 ± 13.46	70.08 ± 11.08	0.546
Height (cm)	162.58 ± 7.00	161.08 ± 6.40	0.291
BMI (kg/m^2^)	27.06 ± 4.75	27.01 ± 4.09	0.901
Sex			
Male	2 (4.6%)^b^	5 (11.9%)	0.240
Female	41 (95.4%)	37 (88.1%)	
Education Education			
Illiterate	7 (16.3%)	1 (2.4%)	0.089
Primary school	7 (16.3%)	7 (16.7%)	
Secondary school	2 (4.6%)	0 (0.0%)	
High school	3 (7.0%)	5 (11.9%)	
Diploma	9 (20.9%)	9 (21.4%)	
University	15 (34.9%)	20 (47.6%)	
Job			
Unemployed	23 (53.5%)	20 (47.6%)	0.529
Employed	20 (46.5%)	22 (52.4%)	
Marital status			
Single	5 (11.6%)	8 (19.0%)	0.399
Married	38 (88.4%)	34 (81.0%)	
BMI Classification			
Normal (18.5–24.9)	15 (35.0%)	11 (26.2%)	0.955
Overweight (25–29.9)	14 (32.5%)	26 (61.9%)	
Obese (≥ 30)	14 (32.5%)	5 (11.9%)	
Physical activity			
Low	31 (72.1%)	25 (59.5%)	0.486
Moderate	22 (27.9%)	17 (40.5%)	
Any multivitamin use			
Yes	25 (58.2%)	16 (38.1%)	0.061
No	18 (41.8%)	26 (61.9%)	

*BMI* Body Mass Index, *CRP* C-reactive protein, *EF* energy/fatigue, *EW* emotional wellbeing, *GH* General health, *hs-CRP* high-sensitive, *MDA* malondialdehyde, *MH* mental health, *P* pain, *PF* physical function, *PH* physical health, *PLA* phospholipase A2, *RE* role limitation due to emotional problems, *RP* role limitation due to physical health, *SF* social function, *SOD* superoxide dismutase, *TAC* total antioxidant capacity

^a^Values are means ± SD

^b^Values are N (%)

^c^Significantly different at p < 0.05 by Independent-Samples T test, Mann–Whitney U test or chi-square test

**Table 2:** Distribution of inflammatory and oxidative stress biomarkers and quality of life among the study participants

Variable	Case group (n = 43)	Control group (n = 42)	P value^a^
hs-CRP (g/mlµ)	4.68 ± 4.36	2.92 ± 4.55	0.011
TAC(mmol/L)	2.46 ± 0.49	2.83 ± 0.82	0.065
MDA (nmol/ml)	2.63 ± 0.71	1.98 ± 0.90	> 0.001
SOD (U/ml)	78.89 ± 37.93	154.25 ± 115.93	> 0.001
PLA2 (pg/ml)	6.81 ± 2.22	4.73 ± 2.97	> 0.001
PF	68.67 ± 2.63^1^	75.89 ± 18.95	0.057
RP	34.44 ± 42.40	60.00 ± 42.44	0.004
RE	37.02 ± 41.57	59.98 ± 41.19	0.010
EF	55.50 ± 18.82	62.88 ± 20.35	0.077
EW	61.51 ± 16.87	67.75 ± 17.42	0.073
SF	62.55 ± 24.57	72.50 ± 24.65	0.033
P	48.5 ± 20.91	79.27 ± 28.38	0.028
GH	51.81 ± 20.91	63.13 ± 20.64	0.011
PH	202.97 ± 85.09	278.30 ± 88.36	0.001
MH	213.44 ± 79.18	264.61 ± 65.87	0.001

Values are means ± SD

*CRP* C-reactive protein, *EF* energy/fatigue, *EW* emotional wellbeing, *GH* general health, *hs-CRP* high-sensitive, *MDA* malondialdehyde, *MH* mental health, *P* pain, *PF* physical function, *PH* physical health, *PLA* phospholipase A2, *RE* role limitation due to emotional problems, *RP* role limitation due to physical health, *SF* social function, *SOD* superoxide dismutase, *TAC* total antioxidant capacity

^a^Significantly different at p < 0.05 by Independent-Samples T test, Mann–Whitney U test or chi-square test

Data were more analyzed to determine the association between serum concentrations of biochemical indices with clinical findings and quality of life by Pearson/Spearman correlation. The analysis also performed in controls concerning the correlation of the biomarkers and the quality of life (Table 3).

**Table 3:** Correlations of scores of inflammatory and oxidative stress biomarkers

Variable	Resting pain	Pain intensity with activity	Night pain	PPT	Pain duration	PF	RP	RE	EF	EW	SF	P	GH	PH	MH
*With clinical findings and quality of life in patient (case) group*^*a*^															
hs-CRP	r = 0.349 (0.171–527)	r = 0.259 (−0.067–0.451)	r = 0.285 (0.089–0.481)	r = 0.269 (−0.070–0.468)	r = 0.283 (0.121–0.445)	r = −0.265 (−0.540–0.010)	r = −0.081 (−0.377–0.215)	r = −0.313 (−0.554–0.072)	r = −0.379 (−0.661–0.097)	r = −0.390 (−0.720–0.060)	r = −0.381 (−0.733–0.029)	r = −0.324 (−0.644–0.04)	r = −0.083 (−0.396–0.230)	r = −0.190 (−0.496–0.116)	r = −0.524 (−0.776–0.272)
	*p = 0.001*	*p = 0.049*	*p = 0.043*	*p = 0.047*	*p = 0.007*	p = 0.079	p = 0.596	*p = 0.036*	*p = 0.010*	*p = 0.008*	*p = 0.010*	*p = 0.030*	p = 0.589	p = 0.212	*p = 0.001*
TAC	r = −0.312 (−0.609–0.015)	r = −0.116 (−0.422–0.190)	r = −0.253 (−0.551–0.045)	r = −0.041 (−0.348–0.266)	r = 0.180 (−0.122–0.482)	r = 0.410 (0.130–0.690)	r = 0.615 (0.373–0.857)	r = 0.123 (−0.182–0.428)	r = −0.012 (−0.319–0.295)	r = −0.104 (−0.410–0.202)	r = 0.253 (−0.044–0.551)	r = 0.228 (−0.072–0.527)	r = 0.415 (0.135–0.695)	r = 0.359 (0.072–0.646)	r = 0.119 (−0.186–0.424)
	*p = 0.037*	p = 0.447	p = 0.093	p = 0.789	p = 0.236	*p = 0.005*	*p < 0.001*	p = 0.421	p = 0.939	p = 0.497	p = 0.093	p = 0.132	*p = 0.005*	*p = 0.016*	p = 0.436
MDA	r = 0.241 (−0.043–0.525)	r = 0.287 (−0.008–0.581)	r = 0.082 (−0.224–0.389)	r = 0.234 (−0.064–0.533)	r = 0.259 (−0.038–0.556)	r = 0.150 (−0.154–0.454)	r = 0.123 (−0.182–0.428)	r = 0.041 (−0.266–0.348)	r = 0.028 (−0.280–0.335)	r = 0.033 (−0.274–0.340)	r = 0.085 (−0.222–0.391)	r = 0.271 (−0.025–0.567)	r = 0.008 (−0.300–0.315)	r = 0.161 (−0.143–0.465)	r = 0.048 (−0.260–0.355)
	p = 0.110	p = 0.056	p = 0.592	p = 0.121	p = 0.086	p = 0.325	p = 0.421	p = 0.790	p = 0.856	p = 0.829	p = 0.580	p = 0.072	p = 0.959	p = 0.291	p = 0.756
SOD	r = −0.184 (−0.457–0.089)	r = −0.076 (−0.383–0.230)	r = −0.101 (−0.407–0.205)	r = −0.029 (−0.336–0.279)	r = 0.277 (−0.072–0.626)	r = 0.077 (−0.229–0.384)	r = 0.029 (−0.279–0.336)	r = 0.124 (−0.181–0.429)	r = 0.120 (−0.186–0.425)	r = −0.114 (−0.420–0.191)	r = −0.033 (−0.340–0.275)	r = 0.133 (−0.172–0.438)	r = 0.057 (−0.250–0.364)	r = 0.077 (−0.229–0.384)	r = 0.057 (−0.250–0.364)
	p = 0.225	p = 0.618	p = 0.507	p = 0.851	p = 0.133	p = 0.614	p = 0.852	p = 0.416	p = 0.433	p = 0.455	p = 0.831	p = 0.383	p = 0.709	p = 0.614	p = 0.709
PLA2	r = 0.263 (−0.020–0.546)	r = 0.131 (−0.165–0.427)	r = 0.140 (−0.131–0.411)	r = 0.025 (−0.238–0.288)	r = −0.074 (−0.373–0.225)	r = −0.137 (−0.474–0.200)	r = −0.383 (−0.604–0.162)	r = −0.314 (−0.544–0.084)	r = −0.140 (−0.453–0.173)	r = −0.152 (−0.436–0.132)	r = −0.086 (−0.384–0.212)	r = −0.328 (−0.560–0.096)	r = −0.233 (−0.498–0.032)	r = −0.310 (−0.488–0.132)	r = −0.210 (−0.587–0.167)
	p = 0.071	p = 0.393	p = 0.360	p = 0.869	p = 0.361	p = 0.369	*p = 0.009*	*p = 0.035*	p = 0.357	p = 0.319	p = 0.576	*p = 0.028*	p = 0.124	*p = 0.038*	p = 0.165
*With quality of life in control (healthy) group*^*a*^															
hs-CRP	–	–	–	–	–	r = −0.178 (−0.470–0.123)	r = −0.108 (−0.399–0.182)	r = −0.265 (−0.559–0.030)	r = 0.001 (−0.305–0.307)	r = −0.423 (−0.700–0.147)	r = −0.203 (−0.502–0.096)	r = −0.295 (−0.600–0.123)	r = −0.071 (−0.399–0.257)	r = −0.222 (−0.515–0.071)	r = −0.351 (−0.637–0.065)
						p = 0.079	p = 0.094	p = 0.077	p = 0.994	*p = 0.004*	p = 0.178	p = 0.058	p = 0.240	p = 0.104	*p = 0.017*
TAC	–	–	–	–	–	r = 0.329 (0.038–0.619)	r = 0.615 (0.372–0.857)	r = 0.444 (0.169–0.720)	r = 0.246 (−0.052–0.544)	r = 0.225 (−0.074–0.525)	r = 0.450 (0.175–0.724)	r = 0.489 (0.221–0.757)	r = 0.618 (0.377–0.860)	r = 0.667 (0.438–0.896)	r = 0.577 (0.326–0.828)
						*p = 0.027*	*p < 0.001*	*p = 0.002*	p = 0.103	p = 0.137	*p = 0.002*	*p = 0.001*	*p < 0.001*	*p < 0.001*	*p < 0.001*
MDA	–	–	–	–	–	r = −0.296 (−0.590–0.003)	r = −0.637 (−0.874–−0.399)	r = −0.314 (−0.606–0.022)	r = −0.280 (−0.575–0.016)	r = −0.510 (−0.775–−0.245)	r = −0.291 (−0.585–0.004)	r = −0.553 (−0.810–−0.297)	r = −0.642 (−0.878–−0.406)	r = −0.697 (−0.918–−0.477)	r = −0.523 (−0.785–−0.261)
						*p = 0.048*	*p < 0.001*	*p = 0.036*	p = 0.063	*p < 0.001*	p = 0.053	*p < 0.001*	*p < 0.001*	*p < 0.001*	*p < 0.001*
SOD	–	–	–	–	–	r = 0.376 (0.091–0.661)	r = 0.681 (0.456–0.906)	r = 0.407 (0.126–0.688)	r = 0.208 (−0.093–0.508)	r = 0.261 (−0.036–0.558)	r = 0.080 (−0.195–0.355)	r = 0.129 (−0.170–0.429)	r = 0.557 (0.326–0.828)	r = 0.716 (0.501–0.930)	r = 0.546 (0.288–0.803)
						*p = 0.011*	*p < 0.001*	*p = 0.005*	p = 0.171	p = 0.084	p = 0.315	p = 0.208	*p < 0.001*	*p < 0.001*	*p < 0.001*
PLA2	–	–	–	–	–	r = −0.328 (−0.619–0.037)	r = −0.381 (−0.665–0.097)	r = −0.290 (−0.584–0.004)	r = −0.203 (−0.504–0.098)	r = −0.339 (−0.928–0.050)	r = −0.548 (−0.805–0.290)	r = −0.424 (−0.702–0.145)	r = −0.412 (−0.692–0.131)	r = −0.486 (−0.754–0.217)	r = −0.532 (−0.793–0.272)
						*p = 0.028*	*p = 0.010*	p = 0.053	p = 0.181	*p = 0.023*	*p < 0.001*	*p = 0.004*	*p = 0.005*	*p = 0.001*	*p < 0.001*

Unadjusted linear regression was used. Values of p < 0.05 are considered as statistically significant

*PPT* pressure pain threshold, *hs-CRP* high-sensitive C-reactive protein, *MDA* malondialdehyde, *PLA2* phospholipase A2, *SOD* superoxide dismutase, *TAC* total antioxidant capacity, *EF* energy/fatigue, *EW* emotional wellbeing, *GH* general health, *MH* mMental health, *P* pain, *PF* physical function, *PH* physical health, *RE* role limitation due to emotional problems, *RP* role limitation due to physical health, *SF* social function

### Discussion

#### Comparison of serum concentrations of inflammatory and oxidative stress indices among participants with and without MPS

In this study, we showed that circulating plasma levels of inflammatory indices of hs-CRP and PLA2 and also MDA as an oxidative stress marker are higher and the plasma level of SOD antioxidant enzyme is lower in patients with MPS in comparison with healthy subjects.

Although serum concentrations of inflammatory and oxidative stress indices have been researched in different pain disorders, data in MPS patients are few. Human studies suggest a probable pathogenic role of the cytokines in pain [19]. Koch et al. [20] revealed the elevated levels of IL-6 in patients with light pain and elevated levels of IL-6 and nitrate/nitrite in severe pain patients. Other studies have shown increased levels of IL-8 pro-inflammatory cytokine in blood and also Cerebrospinal fluid (CSF) of the patients with chronic pain [21], elevated levels of IL-6, IL-1β, and TNF-α in CSF of the patients with complex regional pain syndrome [22] and prominent levels TNF-α in Schwann cells of the patients with painful neuropathies [23]. Nonetheless, Ludwig et al. [24] and Fischer et al. [25] didn’t found any significant differences in terms of serum or CSF concentrations of interleukin-6 and TNF-α and plasma levels of MDA, F2-isoprostanes, and 8-hydroxy-2-deoxyguanosine between patients with or without painful polyneuropathies and Complex Regional Pain Syndrome.

During the inflammatory response, an increase in the amount of oxidative stress also contributes to the pain. Reactive oxygen species (ROSs) and reactive nitrogen species (RNSs) can, directly and indirectly, induce sensitization and activation of nociceptor receptors [26]. The accumulation of ROSs in skeletal muscle contributes to myopathy and contractile dysfunction, both of which are components of MPS [27]. Taken together, these data suggest that complex and multi-mechanism regulatory systems for the role of cytokines in pain are not fully understood.

#### Correlation between serum levels of inflammatory and oxidative stress indices and pain intensity and quality of life among participants with and without MPS

Based on our findings, higher serum concentrations of hs-CRP and lower serum level of TAC was associated with higher pain intensity and duration in patients with MPS. These findings suggest that key symptoms of MPS may partly be the consequence of systemic inflammation.

Pain experimental models established pro-nociceptive properties of cytokines [28]. In the clinical background, the association between CRP (as a substitute for IL-6) and other inflammatory cytokines and pain has been shown in different situations [29–31].

We found that, there were significant and negative correlations between some scales and subscales of quality of life and serum concentrations of hs-CRP and PLA2 in MPS patients. In return, TAC concentration was directly correlated with some scales and subscales of quality of life in the patients. In healthy controls, TAC and SOD concentrations were directly and hs-CRP, PLA2, and MDA concentrations were negatively correlated with scales and subscales of quality of life.

Previous studies have evaluated the association between inflammation and quality of life in other disorders [32–34] and consistent with the results of the present study, the relationship between inflammatory biomarkers and quality of life components in other conditions has been emphasized [35, 36].

The association between mental conditions and the endocrine and immune systems, especially inflammation may be somewhat explained by the hypothalamic–pituitary–adrenal (HPA) axis function. Given that acute psychological stress can induce an inflammatory response [37], stress perception may contribute to the quality of life and may explain some of the present findings. Furthermore, inflammation may disturb the quality of life because of reduced happiness [38]. So, inflammation management is serious for preserving mental health and quality of life.

The findings of this study may theoretically impact clinical practice by suggesting a novel approach to symptom treatment in which systemic inflammation is recognized as a key therapeutic object.

### Conclusion

Patients with MPS had significantly higher concentrations of hs-CRP, MDA, and PLA2 and lower concentrations of SOD in comparison with healthy subjects. In these patients, higher concentrations of hs-CRP and lower concentrations of TAC were associated with higher pain intensity and duration and lower scores of some scales and subscale of quality of life. The serum level of PLA2 was also associated negatively with some scale and physical health subscale scores of quality of life in MPS patients. Additionally, serum concentrations of SOD and TAC were positively and serum concentrations of PLA2, MDA, and hs-CRP were inversely associated with different scale and physical and mental health subscale scores of quality of life in healthy matched controls.

## Limitations

Our study has a relatively small sample size. Additionally, the case–control design limits the capability to causality assessment of how inflammatory and oxidative stress biomarkers implements in predicting MPS. Moreover, the serial examination of biomarker levels for assessing dynamic alterations was not examined. Lack of dietary intake assessment of micronutrients with anti-inflammatory and anti-oxidative properties is another limitation. Furthermore, we did not execute subgroup analysis based on the levels of stress and other psychological elements that may be accountable for the raise in the biomarker concentrations. Finally, we studied a particular group of MPS (i.e., MTrPs the trapezius, the infraspinatus, or the levator scapulae muscles). Thus, external validation may not be applied to other patients with MPS.

## Data Availability

All the necessary data are presented herewith. However if needed, raw data on excel format can be availed on reasonable request from the corresponding author.

## Abbreviations

BMI: Body Mass Index
CRP: C-reactive protein
CSF: Cerebrospinal fluid
EF: Energy/fatigue
EW: Emotional wellbeing
GH: General health
hs-CRP: High-sensitive
IPAQ: International Physical Activity Questionnaire
MDA: Malondialdehyde
MH: Mental health
MPS: Myofascial pain syndrome
MTrPs: Myofascial trigger points
P: Pain
PF: Physical function
PH: Physical health
PLA: Phospholipase A2
RE: Role limitation due to emotional problems
RNSs: Reactive nitrogen species
ROSs: Reactive oxygen species
RP: Role limitation due to physical health
SF: Social function
SOD: Superoxide dismutase
TAC: Total antioxidant capacity
TNF: Tumor necrosis factor
